# User fee exemptions and excessive household spending for normal delivery in Burkina Faso: the need for careful implementation

**DOI:** 10.1186/1472-6963-12-412

**Published:** 2012-11-21

**Authors:** Amal Ben Ameur, Valéry Ridde, Aristide R Bado, Marie-Gloriose Ingabire, Ludovic Queuille

**Affiliations:** 1 International Development Research Centre (IDRC, Ottawa, Canada; 2 Centre de recherche du Centre Hospitalier de l'Université de Montréal, Montréal, Québec (CRCHUM), Canada; 3 Department of Social and Preventive Medicine, Université de Montréal, Montréal, Québec, Canada; 4 Institut de Recherche en Sciences de la Santé (IRSS, Ouagadougou, Burkina Faso

## Abstract

**Background:**

In 2006, the Parliament of Burkina Faso passed a policy to reduce the direct costs of obstetric services and neonatal care in the country’s health centres, aiming to lower the country’s high national maternal mortality and morbidity rates. Implementation was via a “partial exemption” covering 80% of the costs. In 2008 the German NGO HELP launched a pilot project in two health districts to eliminate the remaining 20% of user fees. Regardless of any exemptions, women giving birth in Burkina Faso’s health centres face additional expenses that often represent an additional barrier to accessing health services. We compared the total cost of giving birth in health centres offering partial exemption versus those with full exemption to assess the impact on additional out-of-pocket fees.

**Methods:**

A case–control study was performed to compare medical expenses. Case subjects were women who gave birth in 12 health centres located in the Dori and Sebba districts, where HELP provided full fee exemption for obstetric services and neonatal care. Controls were from six health centres in the neighbouring Djibo district where a partial fee exemption was in place. A random sample of approximately 50 women per health centre was selected for a total of 870 women.

**Results:**

There was an implementation gap regarding the full exemption for obstetric services and neonatal care. Only 1.1% of the sample from Sebba but 17.5% of the group from Dori had excessive spending on birth related costs, indicating that women who delivered in Sebba were much less exposed to excessive medical expenses than women from Dori. Additional out-of-pocket fees in the full exemption health districts took into account household ability to pay, with poorer women generally paying less.

**Conclusions:**

We found that the elimination of fees for facility-based births benefits especially the poorest households. The existence of excessive spending related to direct costs of giving birth is of concern, making it urgent for the government to remove all direct fees for obstetric and neonatal care. However, the policy of completely abolishing user fees is insufficient; the implementation process must have a thorough monitoring system to reduce implementation gaps.

## Background

In order to improve maternal health and achieve Millennium Development Goal 5 (MDG5), African countries introduced different approaches to reduce barriers that limit women’s access to primary health services [1]. Out-of-pocket fees are one of these barriers. They constitute a financial burden, usually associated with catastrophic expenditures that either push already vulnerable households into poverty or worsen their poverty situation [2]. In the early 1980s, many African countries adopted structural adjustment policies that led to the introduction of user fees in the health sector. This required users to pay for delivery-related health services received.

The situation has evolved since then, and today there is a growing shift, globally and particularly in Africa, to reduce financial barriers to health care. There is now a consensus that user fees often constitute a significant barrier to rational service use. They also increase the inequity gap amongst different socio-economic groups in terms of access to primary health care [3]. Due to the adverse effects of user fees on access to basic services, many organizations and governments advocate the removal of these fees. The focus has been on exempting specific services and targeting the most vulnerable [4-6]. Our study will cover a rural region in Burkina Faso, a West African country with high levels of maternal mortality (560/ 100 000 live births; range: 300–950) [7] and widespread poverty (46.4% population living below the poverty line) [8].

### Government interventions

The Government of Burkina Faso is committed to improving access to maternal care. To reduce the maternal mortality and morbidity levels, the government decided in 2006 to subsidize the direct costs of obstetric care (OC) and neonatal services at 80% of the total cost. This would reduce financial barriers and encourage women to make greater use of these services. Beneficiaries pay the remaining 20% of the medical costs at primary health centres (CSPS). Where a normal institutional birth costs 4500 F CFA (≈ 9 US$), the government pays 3600 F CFA (≈7 US$) while the birth mother pays the remaining 20%, or 900 F CFA (≈ 2 US$). The government exemption applies to the total cost of the medical procedures, drugs and consumables, and observation. In addition, the government policy includes the costs for any needed referral by public ambulance to a district or regional hospital in case of a complication. Since 2004, traditional birth attendants have been encouraged to promote medically assisted births rather than assisting in home births [9].

### NGO complementary interventions

In 2008, a German NGO (HELP) funded by the European Commission of Humanitarian Office (ECHO) began work in Burkina Faso to improve access to quality health care for pregnant women. A HELP pilot project covers two rural districts of Sebba and Dori in the northeastern part of Burkina Faso, and offers a full exemption by covering the 20% of costs not covered by the government. The main activities of the NGO are the following: 1) subsidize health care for pregnant women 2) control the exemption system in the pilot districts 3) improve, monitor, and supervise quality of care to pregnant women. Every month, HELP reimburses each CSPS as per their report of real expenditures related to maternal services. Through this intervention, HELP provides added financial protection to households living in the pilot districts by reducing the costs related to an institutional birth.

There are few studies in West Africa examining the effects of user fee exemption policies on the reduction of birth-related expenses and financial protection of households. Thus, the objective of this paper is to investigate the impact of these interventions on the out-of-pocket expenditures of households for non-complicated institutional births in three rural health districts of Burkina Faso. To compare the excessive medical expenses of households receiving the partial exemption (controls) with those receiving the full exemption (cases), we carried out a case–control study in the districts where the NGO-sponsored pilot project resulted in a full exemption from user fees and in a neighbouring district where the government’s partial exemption applied. The findings from this study will allow different health system stakeholders to compare costs and benefits of abolishing user fees versus the current policy of partially subsidizing the costs of institutional births. The ultimate objective of the NGO intervention is to scale it up to the national level through a policy in line with the principle of universal access to primary health care in order to benefit the population accessing health care services. [10]. Thus, stakeholders will need to find strategies to improve successful implementation of exemption models.

## Methods

### Study site

In this study, researchers are analysing the results of a natural experiment. A natural experiment is an empirical study in which the experimental conditions are determined by nature or by other factors out of the control of the evaluators [11]. The study took place in the Sahel Region of Burkina Faso about 300 km from the capital Ouagadougou, which has specific geographical and cultural characteristics. This region has four districts: Dori, Sebba, Djibo and Gorom-Gorom. The first two represent the only districts where the NGO intervened during the study; the evaluators had no control in selecting these districts for the pilot project. In order to select a third district for comparison with Dori and Sebba we examined the level of facility-based delivery (expressed as a percentage of all institutional deliveries) in Gorom-Gorom and Djibo. According to Annuaire statistique 2009 (Ministère de la santé - Secrétariat général, Direction générale de l’information et des statistiques sanitaires), 22% of institutional deliveries in Gorom-Gorom had skilled care, while this proportion was 52.4% in Djibo. Djibo was chosen as the comparison district because its percentage of skilled care was much closer to the proportions observed in the Dori and Sebba districts. Dori and Sebba served as the case districts with a full user fee exemption provided by a pilot project of the NGO HELP, and Djibo as the control district with the government-mandated partial user fee exemption. The table below describes the main characteristics of the three districts (Table 1). 

**Table 1:** **Districts characteristics** (**source: Plans d’Action 2010 des Districts Sanitaires de la Région du Sahel)**

**Characteristics**		**District**		
	**Sebba**	**Dori**	**Djibo**	
Government intervention (partial exemption In 2007)	Yes	Yes	Yes
NGO HELP intervention (total exemption In 2008)	Yes	Yes	No
Official cost of normal delivery to woman*	0 FCFA	0 FCFA	900 FCFA
Total population (2010)	179 819	295 027	389 839
Surface (km2)	6 591	6 920	12 273
Population below the poverty line (%) (2003)	52%	44.6%	37,2%
Main religion represented	Islam	Islam	Islam
Main ethnicity represented	Peulh	Peulh	Peulh
Facility-based delivery (%) (2008)**	47.2%	34.8%	41.0%
Facility-based delivery (%) (2009)**	77.8%	52.6%	52.4%
Facility-based delivery (%) (2010)**	91.2%	59.9%	51.2%
Facility-based delivery (%) (2011)**	96.6%	70,0%	55,0%
Health infrastructure (2010)	1 district hospital 11 CSPS	1 regional hospital 18 CSPS 1 military clinic	1 district hospital 30 CSPS 1 military clinic 3 private health facilities

(*) The official cost of delivery corresponds to « acts, medicines and consumables, and observation » (Ministère de la santé, 2006a).

(**) Annuaire statistique 2008, 2009, 2010, 2011 Ministère de la santé - Secrétariat général, Direction générale de l’information et des statistiques sanitaires.

NGO= Non government organization.

CSPS= Centre de santé et de protection sociale, i.e. “first line health centre”.

F CFA= Franc de la Communauté Financière Africaine.

« facility-based delivery » refers to facility-based childbirths assisted by professionals. According to the Annuaire Statistique where the indicator comes from, it is called in French: Accouchements assistés par du personnel de santé.

### Health centre selection and population sampling

In every district, six health centres were selected for a total of 18 health centres (30% of the total centres). This selection sample was based on two previous studies in the region that reflected situational diversity [12][13]. Only two exceptions were made because of access constraints due to the rainy season at the time of data collection. For these two cases, two other CSPS with similar characteristics were chosen.

The selection sample includes only women who gave birth in health centres and had no complications, as the study focused on expenses related to an institutional birth.

In the full exemption districts (Dori and Sebba), the study population was women who gave birth in health centres two months prior to the start of the study. In the partial exemption district (Djibo), the study population was women who gave birth in health facilities in the six months prior to the start of the study. This longer recall period was needed to gather a large enough sample size because the number of women using facility-based services in that district was very low. At the CSPS level, the sampling was done using maternal registers available in each health centre. A random sample of approximately 50 women per health centre was selected from the registers. The survey size was limited to 50 per health centre due to the limited financial resources available for the study. In Dori and Sebba it was possible to achieve this target. However, in Djibo, the low rate of facility-based births (2010) together with an exceptional number of emigrants made it more difficult to reach our targeted sample size. Thus, the final sample in this district was 270 women rather than 300. The total sample was 870 women who gave birth in 18 health centres. Twenty-one women were excluded from the original sample as they were found to have given birth at home or before reaching the health centre (see Table 2).

**Table 2:** Sampling

**Districts**	**Sebba**	**Dori**	**Djibo**	**Total**
Government intervention (partial exemption)	Yes	Yes	Yes	-
NGO HELP intervention (total exemption)	Yes	Yes	No	-
Total deliveries (N) in 6 health centres1	687*	549*	391**	1627
Random sampling	299	301	270	870
Outliers (home delivery /on the way)	9	8	4	21
Total (n) deliveries selected	290	293	266	849

(1) Source: maternal registers available in health centres Period of delivery: *March 2010- May 2010; **December 2009- May 2010.

### Survey instruments

Survey instruments were developed based on a similar survey conducted in another district of Burkina Faso in 2006 by IMMPACT researchers and in 2010 by VR and AB in Ouargaye [14]. The instruments measure medical household expenditures related to institutional birth at the point of use. The medical expenditures include: user fees, drugs and consumables, laboratory fees, and cleaning products.

Traditional poverty measurements based on consumption or incomes were particularly difficult in the sample district settings. Instead, household characteristics and asset ownership are widely used as indicators of wealth [15]. Consequently, a series of questions related to the specific characteristics of rural households in Burkina Faso was used to gauge household economic status. Questionnaires and consent forms were developed in French and then translated into the local language, Peulh, following the double-translation method. The instruments were pretested in the Dori district in a CSPS not included in the sample. The pre-test assessed the validity of the data-collection instruments and procedures, as well as the sampling procedures. This process identified content and logistical issues that led to revising some of the questions and the data collection process.

### Data collection

Sahel Regional Authorities approved the survey. Fieldwork was conducted over a five-week period starting in May 2010 by six trained local interviewers fluent in Peulh and French. Each interviewer interviewed +/− 50 women over a ten-day period. The household survey questionnaires were administered to all study participants. Respondents gave verbal consent to the interview and were assured of data confidentiality. Two research coordinators carefully supervised data collection during the entire fieldwork.

### Data analysis

Data was input and validation was performed with the double entry method using Epi Data software, and the data set was then converted to SPSS 17 and STATA 11 for analysis. Analysis of variance (ANOVA) was performed to compute means of medical expenses and compare them between districts and other explanatory variables (education, distance, etc.).

A poverty proxy was developed using household data indicators. Through Principal Components Analysis (PCA), households/women were assigned to poverty quintiles [16]. This allows a classification of households from the poorest (rated as 1) to the least poor (rated as 5) in order to show socio-economic differences.

As we did not have information about household consumption and/or income, it is not possible to measure catastrophic expenditures related to births health centres. Instead, we relied upon the concept of "high delivery health care spending per household", equivalent to “excessive spending per household”. It was estimated by analyzing the expenditures dispersion within the two samples (full exemption vs partial exemption group). By using the statistical outlier method (or Tukey method) it was possible to identify the value threshold. Supposing Q1 and Q3 are respectively the first and third quartiles of the distribution of delivery health expenditure of the sample, then a high-spending or outlier household within the group is one for which the value of delivery health expenditure is greater than Q3+k*(Q3-Q1), where k is a constant (varied from 0.5, 1 or 1.5). Having three values for k, rather than one, allows more flexibility in defining the outlier households at different expenditure cut-off points and permits testing the sensitivity of the results. In fact, the smaller k is, the stricter is the approach [17].

### Ethics statement

The Ministry of Health of Burkina Faso examined and approved the ethics component of this research project and authorized the study. Ethical approval was given prior to data collection.

## Results

### Sample characteristics

The socio-economic and demographic status of the sample households is presented in Table 3. The table shows that social, economic, and demographic characteristics are very similar within the three districts. The table shows a difference in the variable “distance from home to health facility”. In fact, women selected from Dori and Djibo lived nearer the health centres than did study subjects from Sebba. Despite this observed difference, the sample characteristics are fundamentally comparable (see Table 3).

**Table 3:** Sample characteristics by districts

**Variables**	**Districts**			
	**Sebba**	**Dori**	**Djibo**	**Total**
	**%**	**%**	**%**	**%**
Age				
15-24	53.8	55.3	56.8	55.2
25-34	35.5	35.8	36.8	36.0
35 and more	10.7	8.9	6.4	8.7
Ethnic group				
Peulh	73.4	86.7	68.0	78.4
Gourmatché	22.4	0.7	0,4	8
Mossi	2.4	4.8	30.1	11.9
Other	1.4	1	1.5	1.3
Matrimonial status				
Single	0	2	0	0.7
(Married) monogamous	82.1	82.3	77.8	80.8
(Married) polygamous	17.9	15.7	22.2	18.5
Education level				
No school	81.4	84.0	91.4	85.4
Literacy	11.4	8.9	4.9	8.5
Primary school	5.9	4.4	2.6	4.4
Secondary and more	1.4	2.7	1.1	1.8
Parity				
Primiparous	28.7	25.9	24.9	26.5
Multiparous (2 to 4)	71.3	75.4	77.5	74.6
High multiparous (5 and more)	0	21.1	12.1	11.1
Distance from home to health facility				
Less than 5km	34.1	61.4	65.0	53.2
5-9 km	37.9	27.6	25.6	30.5
10 km and more	22.1	10.6	9.4	14.1
N/D	5.9	0.3	0	2.1

### Medical expenses distribution

**Figure 1:**
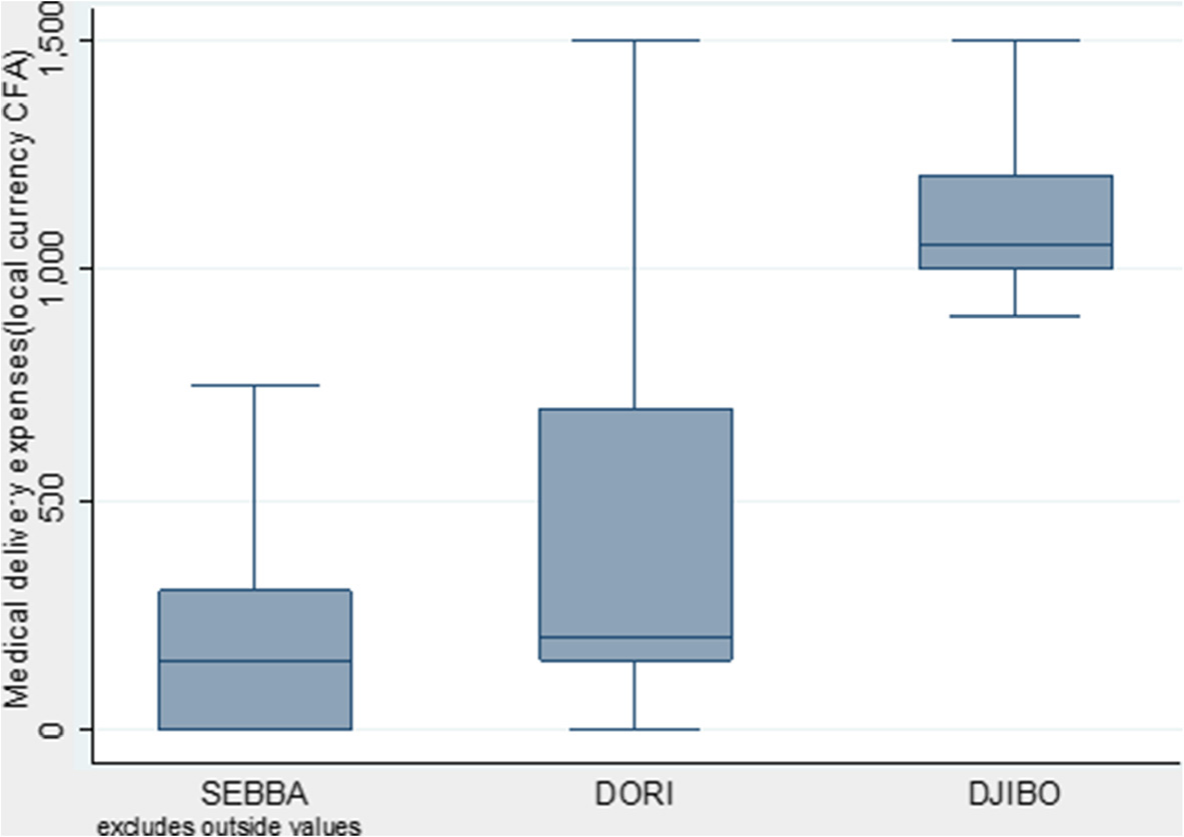
Distribution of total medical delivery expenses in the three districts. Median, interquartile gap and extreme values.

The distribution of the medical expenditures is illustrated in Figure 1. This figure represents Box plot, a visual representation of both central tendency and dispersion of the total medical expenses of the three samples. It simultaneously shows the 25th, 50th (median), and 75th percentile scores, along with the minimum and maximum scores. It is used to compare total medical household expenditures within and between the three districts (see Figure 1).

The figure illustrates significant differences in medical expenses distribution between the three groups. In fact, medical expenses are lower and the distribution is reduced for all the sample women in Sebba, where there is a full exemption. However, in Dori, where there is a full exemption as well, the box plot shows a large distribution of medical expenses. In Djibo, the partial exemption district, the distribution of total medical expenses appears systematically higher than the “fixed price” of 900 F CFA.

To complement the figure, Table 4 describes the medical expenses distribution according to the type of expenditure made by the households. In this context, it is also essential to identify not only the amount but also the nature of expenses at the point of use.

**Table 4:** Detail of medical expenses distribution by district

	**Sebba (1)**				**Dori (2)**				**Djibo (3)**				P value (*)		
	**N**	**Median**	**Q25**	**Q75**	**N**	**Median**	**Q25**	**Q75**	**N**	**Median**	**Q25**	**Q75**	**1vs 3**	**2vs3**	**1 vs 2**
User fees for delivery	2	400	400	400	5	200	200	200	266	900	900	900	0.001	0.001	0.102
Medecine/															
consummable fees (to health professional)	4	237.5	138	650	40	1500	925	1800	11	1000	600	2200	0.297	0.001	0.001
Medecine/															
consummable fees (to pharmacist)	21	100	0	300	22	1000	800	1650	40	1815	1000	2250	0.014	0.419	0.004
Cleaning product fees	205	200	200	200	240	196	150	300	240	150	100	300	0.679	0.016	0.002
Total medical expenses	282	150	0	300	275	200	150	700	266	1075	1000	1275	0.001	0.001	0.002

1= Sebba, 2= Dori, 3= Djibo (*) Differences are considered to be significant when the P value is < 0.05. The median has been measured among the women that paid.

The table shows significant difference in the expenses (median) between the three groups. The first line of user fees shows that in Djibo, women in the partial exemption district had to pay user fees, as expected. However, a few women in Dori and Sebba testified to expenses related to user fees for childbirth, while it should be free for them. In addition, in the three districts, women spent additional amounts on medicines and consumables while all “procedures, drugs and consumables, and observation” are supposed to be included in the official user fee [18]. In fact, a delivery kit includes all the drugs and consumables used in a non-complicated birth at a primary health centre. This kit is part of the government policy implementation plan. Dori seems to be the district with a greater percentage of women paying for medicines and consumables. There is a significant difference of medical expenses between Dori and Sebba, even though they both have the same intervention for full exemption. Finally, the majority of the women had to pay for cleaning products at the point of use. Neither the NGO nor the government exemption included the cost of cleaning products. In both cases, women had to pay for cleaning products used to clean the delivery room after use.

### Excessive spending for households

To assess the impact of health expenditures burden related to an institutional birth, households with excessive health care costs were analysed. This indicator aims to capture empirically the risk of impoverishment related to childbirth and household medical care costs (see Table 5).

**Table 5:** Prevalence of excessive spending within total and partial exemption districts

	**Threshold (Djibo)**	**% of households with excessive spending**							
**K value**		**Sebba**	**Djibo**	**AD (%)**	**RD (%)**	**Dori**	**Djibo**	**AD (%)**	**RD (%)**
k=0.5	1413	1.1	22.5	20.3	100	17.5	22.5	2.8	13.8
k=1.0	1550	-	17.5	18	100	14.9	17.5	3.1	17.2
k=1.5	1688	-	12.0	17.7	100	13.1	12.0	4.6	26.0
	Threshold (Dori)	% of households with excessive spending							
K value		Sebba		Dori		AD (%)		RD (%)	
k=0.5	975	1.1		17.5		21.5		95.3	
k=1.0	1250	-		14.9		17.5		-	
k=1.5	1525	-		13.1		12.0		-	

AD= absolute difference, RD= relative difference.

At any given value of k, the prevalence of households with excessive expenditures is much higher in the partial exemption district (Djibo) compared to Sebba, the only district with almost no excessive spending for households (1.1%; k=0,5). In addition, for two of the three k values, there are no households with excessive expenditure in Sebba where deliveries are free of charge. On the other hand, the comparison between Dori and Djibo is quite unexpected. The percentage of households at risk is very similar in the two districts, even if there are fewer women with excessive expenditures in Dori than Djibo. The comparison between the two districts with the full exemption (Sebba and Dori) demonstrates that Dori has many more households with excessive expenditures than Sebba. Finally, we can observe that for Dori and Djibo, the medical expenses cut-off level is higher for higher values of k. At any given value of k relative and absolute differences are about the same.

### Socio-economic status of household at risk

After looking at the percentage of households with excessive spending in the two groups, table 6 demonstrates the distribution of excessive spending across the socio economic categories (Quintiles). This deeper analysis allows a better understanding of the distribution of the beneficial effect of the user fee related interventions on the poorest and least poor of the region (see Table 6).

**Table 6:** Prevalence of excessive spending by district and quintile

	**Groups**	**Sebba**	**Dori**	**Djibo**
K=0.5	Q1 (poorest)	1.6	18.2	15.9
	Q5 (least poor)	14.5	18.8	20
	Q5-Q1	13.0	0.6	4.1
	Q5/Q1	9.2	1.0	1.3
K=1.0	Q1	0.0	14.5	15.9
	Q5	10.9	18.8	18.8
	Q5-Q1	10.9	4.2	2.9
	Q5/Q1			1.2
K=1.5	Q1	0.0	10.9	13.6
	Q5	0.0	18.8	15.0
	Q5-Q1	0	7.8	1.4
	Q5/Q1	-	1.7	1.1

In the district of Sebba, the poorest seem to reap a greater benefit from the total fee exemption than the less poor. At any level of k, the prevalence of excessive expenses is higher in Djibo and Dori than in Sebba. In addition, the gap between the poor and the less poor related to excessive spending is large in both Dori and Djibo. Finally, we can observe that in the whole sample, the less poor are more at risk of having excessive expenses related to institutional childbirth than the poorest quintile. This gap is more accentuated in Sebba and Dori. This finding implies that the greater the household capacity to pay, the more the household will spend at the facility for medical charges. This last association needs to be further explored in the field.

## Discussion

### The pro-poor benefit of user fee exemption

There is extensive literature on the impact of abolishing user fees on women’s use of health services. For instance, a recent study analysed the effectiveness of the exemption policy and the distribution of its benefits in Burkina Faso. By reducing household expenses, all categories of the population benefited from this policy, including the poorest. However, subsidizing medical costs did not reduce inequalities in the way pregnant women used healthcare. In fact, to improve service utilization by the poorest and reduce expenses more significantly for women living far from health centres, lowering healthcare costs is probably not enough. There are still other financial and geographic barriers to access that need to be removed to increase institutional care for the poorest [14].

There is a consensus that removing or reducing financial barriers increases the number of institutional births. However, fewer studies analyse the risk of further impoverishment in cases where fees have not been totally abolished or where women have to pay other out-of pocket costs at the point of use. Our study shows that total exemption of user fees, such as in Sebba, can be beneficial in reducing excessive expenses for women using delivery care. However, when the additional subsidy was not applied correctly, such as in Dori, household expenditure for direct delivery care costs did not change significantly compared to health centres with only partial exemption. In the Sebba district, where full exemption of user fees was applied, reduction of excessive health expenses was more significant in poor quintiles. It can be argued that, in case of total exemption, the positive impact on poor households would be significant and would contribute to greater equity between the poorest and the richest.

Similar to Ghana’s experience, the elimination of user fees in two of Burkina Faso’s districts benefitted pregnant women regardless of their socio-economic status. Ghana has introduced a similar exemption policy directed at making delivery care free. Findings showed that with its universal application, the policy of abolishing user fees also benefited the poor, thereby addressing the equity issue in their health system. In fact, 18 months after user fees were abolished the greatest increase in health facility use was by the poorest segment of the population [18].

This positive impact of the abolition of user fees on the health care seeking behaviour and on the out-of-pocket expenditures observed in Ghana should encourage Burkina Faso’s Government to expand the full exemption pilot project to the national level.

In a country where almost 46% of the population lives below the threshold of absolute poverty [8], it is important to implement poverty reduction strategies. Full user fee exemptions could improve access to institutional birthing facilities and reduce poverty by decreasing excessive medical expenses. In fact, we already observed that access to facility-based delivery improves dramatically with the reduction of user fees. In three years, institutional deliveries increased by 35 to 50 percentage point in Sebba and Dori and only by 14 percentage point in the control district (Djibo) (see Table 4). Scaling up the full user fee exemption to the national level would certainly broaden its impact.

### Implementation gap

Results in the three districts show inadequate implementation of the user-fee exemption, especially for Dori and Djibo. In fact, respectively 21% and 19% of the women are still paying for drugs and consumables, which should be covered under both the government’s and NGO’s exemptions program. During the household surveys, women reported that they were still paying for items that should be free, such as gloves, syringes and drugs. In addition, in Dori, women are still facing out-of-pocket expenses whereas all the births are supposed to be “free” to the user. When these results were shared in a workshop with different stakeholders in November 2010, health professionals from the region gave some explanations for the added expenses. The main one arising from the discussion was the lack of understanding and confusion regarding both exemption programs [19]. In cases where the nurses and midwives were unclear about the procedures and products included in the subsidies, the patient would end up paying. A second explanation suggested by the staff for the cost of drugs and consumables charged to users was a shortage of products in the health centre pharmacy. In some remote health centres, the pharmacy management was seen as a major challenge, forcing the women to buy the required products for the non-complicated delivery from a private pharmacy. The hypothesis given by health workers about a shortage of products needs to be verified with more qualitative research. However, a similar study in another district (Ouargaye), demonstrated that supply shortages in health centre pharmacies were very rarely involved in the additional expenses incurred by the women. Occasionally, because of the absence of the community pharmacy manager, health workers send patients to a private pharmacy in the village. The study, conducted in a single district, confirmed, measured, and explained why the flat fee was not respected when the policy was implemented. Because this situation has been shown to exist in other districts of the country, it is reasonable to believe that it is a national problem.

The role of drug supply shortages was not really confirmed. However, the combination of three factors: i) products not included in the delivery kit, ii) lack of understanding of the exemption policy and iii) informal charges by health workers; explains part of the implementation gap of the policy at the local level. Nonetheless, more qualitative research is needed to understand all the plausible causes of this gap [20].

Information about the specific components of the exemption policy remains fragmented and uncertain. These problems of implementation and policy understanding are confirmed in two other studies in Burkina Faso [21]. In Nouna and Ouargaye, two regions where the national exemption policy has been evaluated recently, the level of medical expenses is very similar to those in the partial exemption district of this study, Djibo [22]. The medical expenses are usually higher than the designated user fees (900 F CFA), such as in Djibo where 50% of women (median) declared paying more than 1075 F CFA. The policy implementation gap appears to have more severe consequences in Dori and Djibo because the amount paid is much more than the one in Sebba. It represents at least 4 days of earnings for those who are living below the poverty line (46% of the population). This difference between Sebba and Dori could be explained by the fact that Sebba has a Chief Medical Officer with better leadership to implement an effective monitoring and control system to more effectively implement the total exemption policy. A study in Burkina Faso shows how the leadership of the District Chief Medical Officer (DCMO) could affect the performance of the District [23]. This seems to be the case in Dori and Sebba. For example, in 2010 the DCMO of Sebba was able to find a scholarship to obtain a master’s degree and in 2012 the DCMO of Dori was transferred by the administration, reflecting that all health indicators are worse in Dori than Sebba over the last five years (see Table 4). However, further qualitative studies must be done to understand in depth this difference between the two districts. For example, it is crucial to analyse how the NGO monitoring and evaluation system responded to the different leadership in both districts. Finally, the results show that the policy instrument of the abolition of user fees is not enough; the implementation process as well as a thorough monitoring system to reduce the risk of implementation gap are essential [24,25].

In addition, in Burkina Faso, the fixed-rate reimbursement of services represents a profound change to health administration practices, designed to reduce the work burden of health professionals. However, the administration in general in Burkina Faso is more bureaucratic and demands detailed proof for every health service delivered, even with a fixed-rate reimbursement system. This lack of alignment resulted in fixed-rate reimbursement under an actual-cost accounting system. Thus, one important weakness is that the accounting control system is not set up for fixed-rate reimbursement, and discussions are on-going to return to a system based on real expenses, as the actual costs for births are less than the fixed-rate reimbursement. Thus, the implementation gap can also be explained by this administrative conflict between practice and accountability. In fact, health professionals have a financial interest in reducing the real amount of used input to make a profit on the fixed-rate received for the service. Furthermore, despite relatively tight administrative controls, it seems that health workers have figured out how to take advantage of the system [23,26]. It demonstrates once again the necessity to review policy instruments to improve its effectiveness.

The process of policy development and implementation has itself an important influence on effective implementation. In fact, the potential for a “no user fee” policy to translate into reduced mortality and morbidity for mothers and babies depends fundamentally on the effectiveness of its implementation. The NGO implemented a system of monitoring and control of the exemption system in all the health centres in Dori and Sebba. They also supported health centres in running and maintaining their facilities, by building the capacity of the healthcare and administrative staff as well as community health management committees. The NGO also initiated activities to raise community awareness of maternal health-related issues. They use a reimbursement form based on lump sum per delivery that coincides with a flat rate of 900 F CFA. However, findings demonstrate that there is a need for a strong monitoring and evaluation component to address problems related to partial and full policy implementation at the local and national levels, including effective and thorough dissemination of the policies to the communities and health workers [26].

### Strengths and limitations

The adequacy of the study design and the degree of control exercised in the data gathering enhanced the internal validity. The random assignment of the sample and the control of external variables within the districts ensured the analysis of the change in the dependent variable (household expenses). In addition, at any given value of k, findings show the same type of association between the percentage of households with excessive spending and the group affiliation. This homogeneity enhances the reliability of the findings.

One limitation of this study is the potential for recall or memory bias. These measure biases can occur while collecting information about the extent and types of out-of-pocket expenditures by women/households for non-complicated institutional birthing services in the selected health centres. In order to reduce this type of systematic error regarding childbirth expenditures, researchers attempted to limit the period between interviews (May 2010) and time of birth to a maximum of 2 months. However, in cases where total number of deliveries during this period was not sufficient (< 50 women) the period of selection was extended up to 6 months prior to May 2010. This date extension was made only for the comparison district sample.

Another limitation is the lack of data in the districts of Dori and Sebba before the HELP intervention in 2008. In fact, we do not possess the information about the household expenses related to institutional delivery prior to the HELP pilot. Such data would have increased the internal validity of the study. Thus, in a context of natural experimentation, the case–control design is the best design to conduct such study.

The partial exemption district also had some limitation when it comes to analysing the istricts characteristics (Table 4). However, choices of districts for comparison were very limited. Therefore, there are challenges to the evaluation of public health policy interventions as natural experiments because of the lack of control over the study conditions [11]. To reduce this challenge as much as possible, Djibo, which has characteristics most like to Dori and Sebba, was chosen for the study.

## Conclusions

The results of this study are particularly encouraging. They show that the abolition of payment for facility-based births is a solution that is not only equitable, but also potentially useful in reducing the proportion of women for whom excessive medical expenses represent a serious risk to their household well-being. However, the implementation gap in Dori is a lesson to keep improving the monitoring system related to medical expenses at the point of use. Furthermore, additional research is needed to understand factors associated with the “success” of total exemption intervention in Sebba. For example, the leadership of the District Medical Officer must be studied as well as positive and negative incentives that may be used to allow effective application of the exemption at health centre level.

During the study, the government’s partial exemption was based on fixed-rate reimbursement (lump sum flat fee). However, for uncomplicated births, the fixed rate costs were never compared to actual costs of care delivery. The government set the cost for an uncomplicated birth at 4500 F CFA. This included the cost of the delivery kit (drugs and consumables). Women thus have to pay 900 F CFA for uncomplicated births (20%). These expenses were supposed to be included in the national budget for the period 2006–2015, evidence of a real political commitment to reducing maternal mortality and morbidity. However, many studies demonstrated that the fixed rate fee structure is higher than the real cost of uncomplicated births (gap of −17 to −27 between 2006 and 2008). Consequently, abolishing the 900 FCFA payment as promised by the Head of State in February 2010 appears feasible, as long as mechanisms are in place to eliminate informal charges. Indeed, the district of Kaya abolished the 900 FCFA payment without any NGO assistance by using its decentralized credits mechanisms. Furthermore, since 2011 in Djibo and 2012 in Gorum-Gorum the local municipalities have paid the 900 F CFA user fees, showing that political will At local level can reduce financial barriers. Full exemption for all from point-of-service user fees should be considered in order to move toward universal coverage [27,28]. The President of Burkina Faso is therefore accurate when he stated in 2010 that he would embark on completely removing the direct payment for facility-based births. This decision has yet to be implemented, while this study shows how it would be beneficial to women. Therefore, it is now urgent to abolish user fees for births because the State has the financial means to do so. Furthermore, international donors should also reorient their interest on achieving universal health coverage by supporting government implementing total abolition of user fees as an effective strategy toward equity and poverty reduction in developing countries. At the same time, the State must find ways to ensure effective implementation of its policies by making sure that they are clearly understood and by establishing accountability mechanisms. In fact, this study demonstrates clearly that the policy instrument of the abolition of user fees is not enough; the implementation process is essential and needs a thorough monitoring system to reduce the risk of implementation gaps [11].

## Competing interests

The authors declare that they have no competing interests.

## Authors' contributions

VR and LQ were in charge of the original study design. VR, AB and ABA designed the data collection tools. AB, ABA and LQ were responsible for data collection. AB, ABA, MGI and VR conducted the data analysis. All authors contributed to the interpretation of the results. ABA, MGI and VR wrote the manuscript with contributions from all authors. All authors had full access to all of the data (including statistical reports and tables) in the study and can take responsibility for the integrity of the data and the accuracy of the data analysis. All authors read and approved the final manuscript.

## Pre-publication history

The pre-publication history for this paper can be accessed here:

http://www.biomedcentral.com/1472-6963/12/412/prepub
